# Fenofibrate plus statin and ASCVD risk by triglyceride-rich lipoprotein cholesterol in 70,000 patients

**DOI:** 10.1016/j.jlr.2026.101024

**Published:** 2026-03-23

**Authors:** Youngwoo Jang, Byung Jin Kim, Yong Whi Jeong, Dae Ryong Kang, Kwang Kon Koh, Børge G. Nordestgaard, Seung Hwan Han

**Affiliations:** 1 Department of Cardiology, Gachon University Gil Medical Center, Incheon, South Korea; 2 Gachon University, College of Medicine, Incheon, South Korea; 3 Division of Cardiology, Department of Internal Medicine, Kangbuk Samsung Hospital, Seoul, South Korea; 4 Department of Medical Informatics and Biostatistics, Graduate School, Yonsei University, Seoul, South Korea; 5 Department of Precision Medicine, Wonju College of Medicine, Yonsei University, Wonju, South Korea; 6 K-Heart Clinic & Lab, Incheon, South Korea; 7 Department of Clinical Biochemistry, Copenhagen University Hospital – Herlev and Gentofte, Herlev, Denmark; 8 Department of Clinical Medicine, Faculty of Health and Medical Sciences, University of Copenhagen, Copenhagen, Denmark

**Keywords:** cholesterol, lipoproteins, myocardial infarction, statins, triglycerides

## Abstract

Fenofibrate may offer cardiovascular benefit in statin-treated individuals with elevated triglyceride-rich lipoprotein cholesterol (TRL-C), a measure reflecting cholesterol carried in triglyceride-rich lipoproteins, yet its role remains uncertain. We investigated whether fenofibrate added to statin therapy is associated with lower risk of atherosclerotic cardiovascular disease (ASCVD), particularly in individuals with high TRL-C. We analyzed 67,662 statin-treated adults without baseline ASCVD from the Korean National Health Insurance Service cohort. Participants were stratified by median TRL-C (26 mg/dl), and propensity score matching was performed for fenofibrate users and non-users within each stratum. The primary outcome was incident ASCVD, defined as a composite of coronary artery disease, ischemic stroke, and cardiovascular death. Over a mean follow-up of 87 months, fenofibrate use was associated with lower ASCVD risk in individuals with TRL-C ≥ median (adjusted hazard ratio [HR_adj_] 0.76; 95% confidence interval [CI] 0.59–0.99) but not in those with TRL-C < median after matching. The association remained consistent in multiple sensitivity analyses and was most pronounced in individuals with the highest TRL-C quartile (P_interaction_ = 0.04). A significant benefit was also observed in individuals with non–high-density lipoprotein cholesterol (Non-HDL-C) ≥140 mg/dl, with HR_adj_ of 0.68 (95% CI 0.49–0.94) for non–HDL-C 140–172 mg/dl and 0.68 (95% CI 0.46–0.99) for non–HDL-C 172–199 mg/dl. In this nationwide cohort, fenofibrate use was associated with 24% lower ASCVD risk with elevated TRL-C despite statin therapy. These findings suggest that TRL-C may help identify individuals who derive greater benefit from fenofibrate therapy.

## Graphical abstract

Fenofibrate added to Statin Therapy in Individuals with High and low TRL Cholesterol in Primary Prevention. Matched, propensity score matched.

**Figure:**
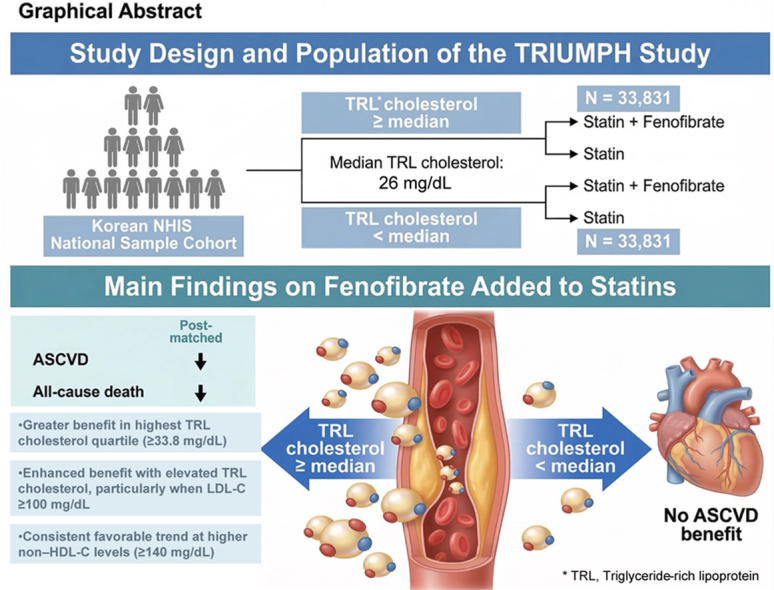

Atherosclerotic cardiovascular disease (ASCVD) remains the number one cause of morbidity and mortality worldwide, with statin therapy being the primary treatment approach for ASCVD prevention. (1, 2) Despite the effectiveness of statins in lowering low-density lipoprotein (LDL) cholesterol and reducing ASCVD risk, substantial residual risk persists, highlighting the need for additional therapies.

A key driver of residual ASCVD risk is elevated triglyceride-rich lipoprotein (TRL) cholesterol. (3, 4, 5, 6, 7, 8, 9, 10, 11, 12, 13, 14, 15, 16, 17) In contemporary literature, the related term remnant cholesterol is increasingly used to describe cholesterol carried in atherogenic TRLs. Although TRL cholesterol and remnant cholesterol are not strictly identical, they substantially overlap in fasting samples. TRL cholesterol broadly includes the cholesterol content of very-low-density lipoproteins (VLDL), intermediate-density lipoproteins (IDL), chylomicrons, and chylomicron remnants, whereas remnant cholesterol more specifically refers to the cholesterol content of remnant particles. (18, 19, 20) Because chylomicrons and chylomicron remnants are negligible in the fasting state, calculated remnant cholesterol [total cholesterol − high-density lipoprotein (HDL) cholesterol − LDL cholesterol] largely reflects fasting TRL cholesterol. Therefore, in the Korean national health screening system, where lipid levels are routinely measured in the fasting state, TRL cholesterol may be considered a practical proxy for calculated remnant cholesterol. (18).

Nevertheless, prior studies have not specifically targeted individuals at high ASCVD risk due to elevated TRL cholesterol using therapies known to reduce these atherogenic lipoproteins. Fibrates are one such class of agents, and in the absence of statin therapy they have been shown to reduce ASCVD risk, particularly among individuals with elevated TRL cholesterol, typically reflected by high triglyceride levels. (6, 21, 22, 23, 24) However, the role of fibrates in statin-treated individuals remains unclear. Fenofibrate may be particularly relevant in this setting because it lowers TRL cholesterol without causing a concurrent increase in LDL cholesterol. (25) In both the FIELD (Fenofibrate Intervention and Event Lowering in Diabetes) (24) and ACCORD (Action to Control Cardiovascular Risk in Diabetes) trial (26), benefit appeared to be concentrated in individuals with high TRL cholesterol, as reflected by higher triglyceride levels. However, the association between fenofibrate and ASCVD risk across different baseline TRL cholesterol levels has not been systematically evaluated in either randomized controlled trials or observational cohorts. We therefore tested the hypothesis that fenofibrate added to statin therapy is associated with lower ASCVD risk in individuals with high TRL cholesterol.

## Materials and methods

### Data source and study population

We analyzed the National Health Insurance Service (NHIS)-National Sample Cohort (NHIS–NSC) of the Korean NHIS. (27, 28, 29) The presently used database was named the TRIUMPH (Triglyceride-RIch lipoprotein cholesterol-gUided fenofibrate therapy in prIMary Prevention among patients with Hyperlipidemia) cohort, which included a sample of 1 million individuals who maintained health insurance and were beneficiaries of medical benefits in Korea; all residents and foreigners staying in Korea are registered to the NHIS. The NHIS–NSC is a representative sample of approximately 2% of the Korean general population. The database contains a de-identified research dataset including sociodemographic information, disease diagnoses, outpatient and inpatient records, therapeutic procedures, drug prescriptions, health examination results, and data on deaths collected by the National Statistical Office, such that the date and cause of death can be determined. A detailed description of these data has been reported elsewhere. (27).

**Fig. 1:**
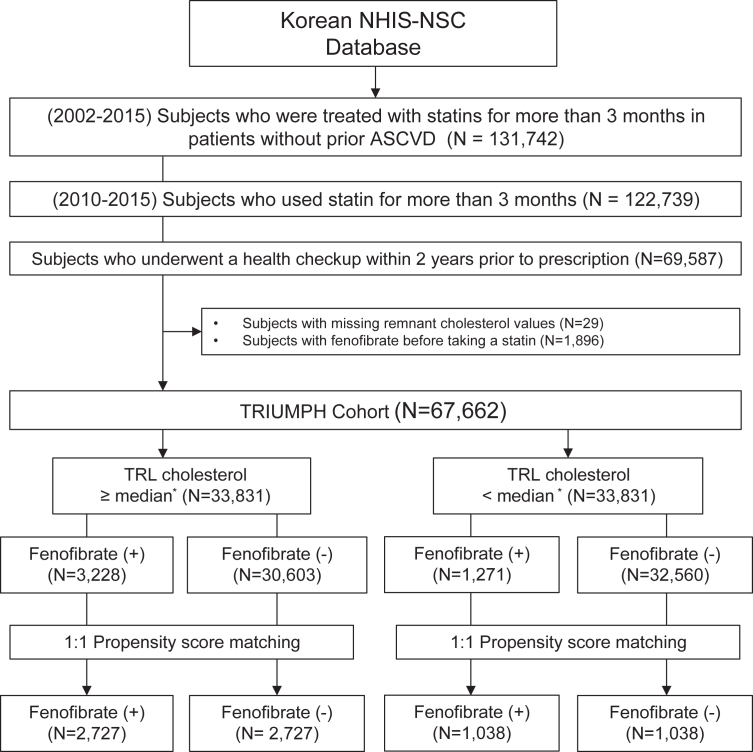
Flow diagram for selection of study participants. NHIS-NSC, National Health Insurance System – National Sample Cohort; ASCVD, atherosclerotic cardiovascular disease; TRIUMPH, Triglyceride-RIch lipoprotein cholesterol-gUided fenofibrate therapy in prIMary Prevention among patients with Hyperlipidemia; matched, propensity score matched. ∗ Median TRL cholesterol: 26 mg/dl.

We conducted a cohort study from 2002 through 2019 to examine the association between fenofibrate therapy added on to statin use (for more than 3 months) with the incidence of ASCVD, that is, in individuals without a history of previous ASCVD and thus in the primary prevention setting (Fig. 1); we used this entire period to identify both ASCVD events before and after entry into the statistical analyses of all participants. As the main participants in the statistical analyses, statin-naïve individuals between 2010 and 2015 were selected to secure sufficient time to capture ASCVD events both before and after study entry and to minimize potential confounding effects due to older data. As health checkups are conducted every two years for every Korean individual, we selected those who had undergone a checkup within 2 years prior to the prescription of statins for the evaluation of TRL cholesterol levels and any lipid-lowering therapy. Individuals with missing data or those who were treated with fenofibrate before statin initiation were excluded. This study was approved by the Institutional Review Board of Gachon University Gil Medical Center, Incheon, Korea (GCIRB2022-297). Written informed consent was waived, as this is a retrospective study of de-identified administrative data. This study was conducted in compliance with the principles outlined in the Declaration of Helsinki (2013).

### Fenofibrate and statin therapy

We selected patients who had been on statin therapy for more than 3 months. Among these, individuals who also received fenofibrate therapy were defined as those who had been prescribed fenofibrate for at least 3 months. To ensure all participants were consistently on statins and fenofibrate throughout follow-up, we utilized NHIS-NSC data, which records the dates and durations of drug prescriptions. The index date for start of follow-up was set at more than 3 months after taking statins. Individuals were considered exposed if they had been prescribed fenofibrate at least once daily for ≥3 months, irrespective of formulation, including tablets (160 mg), extended-release capsules (250 mg), and standard capsules (130 mg or 200 mg). Because direct measures of medication adherence were not available, exposure was defined based on prescription records, and individuals remained in their initially assigned exposure group regardless of subsequent discontinuation. The percentage of drug administration during follow-up was calculated as the ratio of total prescribed days to total follow-up days.

### TRL cholesterol and follow-up lipid measurements

TRL cholesterol was defined as total cholesterol minus HDL cholesterol minus LDL cholesterol and represents the cholesterol content carried within TRLs, including VLDL and IDL, in the fasting state. Although this calculation is mathematically identical to the commonly used formula for calculated remnant cholesterol, we refer to this measure as TRL cholesterol throughout the manuscript to accurately reflect the lipoprotein fraction evaluated in this study. (18) In the Korean National Health Insurance System health examination program, lipid profiles are routinely measured after overnight fasting. Under fasting conditions, chylomicrons and chylomicron remnants are negligible, and calculated remnant cholesterol (total cholesterol minus LDL cholesterol minus HDL cholesterol) largely reflects cholesterol carried in TRLs. Accordingly, TRL cholesterol derived using this calculation is often used as a practical approximation of fasting TRL-related cholesterol in large-scale epidemiologic studies. (30, 31).

Among the several LDL cholesterol estimating methods, we used the Martin-Hopkins method, which is known for its superior accuracy over the Friedewald equation. (32, 33, 34) Lipid profiles of all individuals were required to be measured after overnight fasting. Follow-up lipid measurements were available from periodic national health examinations. Cumulative lipid parameters were defined as the change in lipid levels over time, calculated as the difference between baseline and follow-up values for LDL cholesterol and TRL cholesterol. Follow-up lipid levels were defined as the mean of all available lipid measurements obtained after baseline, excluding the baseline value, among individuals with at least one follow-up measurement during the study period.

### Clinical follow-up and endpoints

The primary outcome was incident ASCVD. ASCVD was defined as a composite of coronary artery disease, ischemic stroke, and ischemic cardiovascular death. Coronary artery disease included myocardial infarction and angina pectoris (ICD-10 I20–I25) requiring hospitalization for ≥2 days with relevant procedure codes. Ischemic stroke was defined using ICD-10 codes I63–I64. Hemorrhagic stroke (ICD-10 I60–I62) was excluded. Cardiovascular death was defined as death attributable to atherosclerotic cardiovascular causes and was identified using ICD-10 codes I20–I25 and I63–I64. Deaths by non-atherosclerotic causes were excluded, including rheumatic heart diseases (I00–I02, I05–I09), hypertensive diseases (I10–I15), pulmonary heart disease and diseases of pulmonary circulation (I26–I28), and other non-atherosclerotic forms of heart disease (I30–I52).

Coronary artery disease was a composite of myocardial infarction and angina pectoris; coronary artery disease was identified as International Classification of Diseases, Tenth Revision (ICD-10) codes I20-I25 during hospitalization for ≥ 2 days, including procedure codes such as coronary angiography, percutaneous coronary intervention, or coronary artery bypass surgery. Stroke was defined as I60–I64 during hospitalization for ≥ 2 days, including insurance claims made for brain magnetic resonance imaging or brain computed tomography. (28) Individuals who developed ASCVD within 3 months of taking statins were excluded to minimize reverse causality in the primary analyses; longer exclusion windows were explored in sensitivity analyses. The last follow-up date was December 31, 2019.

### Covariables and comorbidities

Body mass index was estimated as body weight (kg) divided by height squared (m^2^). Income level was calculated based on the insurance owner’s income and classified into tertiles. Hypertension was identified according to I10 registered diagnoses. Diabetes mellitus was type 2 diabetes (E11–14) with a fasting plasma glucose level ≥ 126 mg/dl or at least one claim per year for the prescription of anti-diabetic drugs. (35) According to ICD-10 codes, chronic kidney disease was defined as N18, atrial fibrillation as I48, and heart failure as I50.

### Statistical analysis

Categorical variables were described as numbers and percentages, and continuous variables were summarized as means ± standard deviations. To compare the baseline characteristics between groups, independent *t* test or Wilcoxon rank-sum test was used for continuous variables, and a chi-square test or Fisher’s exact test for categorical variables. Participants on statins were divided into four groups based on median TRL cholesterol levels and the presence or absence of fenofibrate treatment.

To understand the robustness of the finding for the main analyses, we performed three parallel tests: in the entire cohort of 67,662 individuals before matching, in 7,530 individuals after matching, and in the entire cohort after multivariable adjustment. Additionally, sensitivity analyses were conducted excluding ASCVD events occurring within the first 12 and 24 months of follow-up to minimize reverse causality.

Propensity score matching (referred to as matching) was done between those with and without fenofibrate therapy (Fig. 1). Covariates for calculating propensity scores included age, sex, body mass index, income, hypertension, diabetes, chronic kidney disease, atrial fibrillation, heart failure, fasting blood glucose, total cholesterol, HDL cholesterol, triglycerides, LDL cholesterol, and TRL cholesterol. These variables were selected due to their direct association with ASCVD. Propensity scores were derived using logistic regression to estimate the probability of fenofibrate treatment, based on these covariates, and individuals were matched 1:1 with a calliper width of 0.25 to ensure close similarity between the fenofibrate-treated and untreated groups. (36, 37) After matching, continuous variables were compared using paired sample t-tests or Wilcoxon signed-rank tests and categorical variables using McNemar’s test.

Cumulative ASCVD incidence rates were estimated by the Kaplan–Meier survival curve, and equality was compared using the log-rank test. Cox proportional hazard regression models were used to estimate the association of fenofibrate use at baseline with the risk of ASCVD and all-cause mortality during follow-up, with or without multivariable adjustment for age, sex, body mass index, hypertension, diabetes, income tertile, cumulative LDL cholesterol, and cumulative TRL cholesterol. Covariates that were associated with ASCVD with *P* < 0.05 were selected as adjustment variables. Absolute risk reduction (ARR) and number needed to treat (NNT) were estimated from cumulative incidence curves derived from Kaplan–Meier analyses. ARR was calculated as the absolute difference in cumulative incidence of ASCVD events between statin-treated individuals with and without fenofibrate at prespecified follow-up time points. NNT was calculated as the reciprocal of ARR (1/ARR). ARR and NNT were presented descriptively to illustrate the magnitude of the absolute treatment effect and were not used for hypothesis testing. All sensitivity and subgroup analyses were performed by multivariable adjustment. All statistical analyses were conducted using SAS version 9.4 (Cary, NC, USA) and R 4.3.1 (Institute for Statistics and Mathematics, Vienna, Austria).

## Results

### Baseline characteristics

From among 1 million individuals, we identified 69,587 statin-treated individuals without baseline ASCVD from the Korean NHIS–NSC database (Fig. 1). At baseline, the mean age was 60 ± 11 years and body mass index 25 ± 3 kg/m^2^, while 75% had hypertension and 67% diabetes (Table 1). Median LDL and TRL cholesterol levels were 143 and 26 mg/dl. The mean follow-up duration was 87 ± 26 months. The use of fenofibrate was observed in 7% of the individuals with an average duration of 21 ± 21 months during the cohort period. Most individuals (93%) were on low or moderate-intensity statins.

**Table 1:** Baseline characteristics of the total TRIUMPH cohort

Characteristics	Total (N = 67,662)
**Demographic data**	
Age (years)	60 ± 11
Male sex, n (%)	29,064 (43)
Body mass index (kg/m^2^)	25 ± 3
<18.5, n (%)	805 (1)
18.5-24.9, n (%)	35,501 (52)
25.0-29.9, n (%)	27,102 (40)
>30, n (%)	4,254 (6)
Income	
1^st^ tertile, n (%)	14,929 (22)
2^nd^ tertile, n (%)	23,830 (35)
3^rd^ tertile, n (%)	28,903 (43)
Hypertension, n (%)	50,952 (75)
Diabetes, n (%)	45,627 (67)
Chronic kidney disease, n (%)	2,407 (4)
Atrial fibrillation, n (%)	3,651 (5)
Heart failure, n (%)	7,474 (11)
Follow-up duration (months)	87 ± 26
**Laboratory data**	
Fasting blood glucose (mg/dl)	109 ± 36
Total cholesterol (mg/dl)	224 ± 49
Triglycerides (mg/dl)	164 ± 122
Median triglycerides (mg/dl)	137
LDL cholesterol (Martin-Hopkins)	140 ± 50
Median LDL cholesterol (mg/dl)^a^	143
LDL cholesterol (Friedewald)	136 ± 54
HDL cholesterol (mg/dl)	55 ± 24
TRL cholesterol (mg/dl)^a^	29 ± 15
Median TRL cholesterol (mg/dl)	26
**Medication**	
Fenofibrate administration, n (%)	4,499 (7)
Fenofibrate duration (months)	21 ± 21
Statin	
Low/moderate intensity, n (%)	62,640 (93)
High intensity, n (%)	5,022 (7)
Plus ezetimibe, n (%)	5,939 (9)

Continuous variables were summarized as means ± standard deviations, except where shown as median values related to skewed distributions of lipid values.

TRIUMPH, Triglyceride-RIch lipoprotein cholesterol-gUided fenofibrate therapy in prIMary Prevention among patients with Hyperlipidemia; LDL, low-density lipoprotein; TRL, Triglyceride-rich lipoprotein; HDL, high-density lipoprotein.

a Martin-Hopkins equation was used for LDL cholesterol calculation.

### Matching by baseline characteristics

After excluding individuals who took fenofibrate before a statin or those with missing values, we divided participants into 33,831 and 33,831 individuals with TRL cholesterol at or above and below the median of 26 mg/dl; in those at or above the median, 3,228 (10%) and 1,271 (4%) took fenofibrate. To balance discrepancies in baseline characteristics between individuals with and without fenofibrate treatment in those at or above and below the median for TRL cholesterol, we performed matching based on propensity scoring ending with 2,727 and 1,038 individuals on fenofibrate (with matching number of individuals without fenofibrate use) in the two TRL cholesterol groups (Fig. 1). After the 1:1 matching, there was no difference in baseline characteristics between individuals with and without fenofibrate use in both TRL cholesterol groups (Table 2).

**Table 2:** Post-matched characteristics based on TRL cholesterol levels

Characteristics	TRL Cholesterol ≥ median				TRL Cholesterol < median			
	Total (N = 5,454)	With Fenofibrate (N = 2,727)	Without Fenofibrate (N = 2,727)	*P*	Total (N = 2,076)	With Fenofibrate (N = 1,038)	Without Fenofibrate (N = 1,038)	*P*
Demographic data								
Age (years)	56 ± 10	56 ± 10	56 ± 10	0.78	59 ± 10	59 ± 10	59 ± 10	0.94
Male sex, n (%)	3,196 (41)	1,129 (41)	1,129 (41)	1.00	918 (44)	459 (44)	459 (44)	1.00
Body mass index (kg/m^2^)	26 ± 3	26 ± 3	26 ± 3	0.85	25 ± 3	25 ± 3	25 ± 3	0.87
				1.00				1.00
<18.5	4 (0)	2 (0)	2 (0)		12 (0)	6 (0)	6 (0)	
18.5–24.9	2,204 (40)	1,102 (40)	1,102 (40)		1,164 (56)	582 (56)	582 (56)	
25.0-29.9	2,792 (51)	1,396 (51)	1,396 (51)		822 (40)	411 (40)	411 (40)	
>30	454 (9)	227 (9)	227 (9)		78 (4)	39 (4)	39 (4)	
Income				1.00				1.00
1_st_ tertile	1,172 (21)	586 (21)	586 (21)		452 (22)	226 (22)	226 (22)	
2_nd_ tertile	1,956 (36)	978 (36)	978 (36)		764 (37)	382 (37)	382 (37)	
3_rd_ tertile	2,326 (43)	1,163 (43)	1,163 (43)		860 (41)	430 (41)	430 (41)	
Hypertension, n (%)	4,342 (80)	2,171 (80)	2,171 (80)	1.00	1,938 (83)	969 (83)	969 (83)	1.00
Diabetes, n (%)	4,124 (76)	2,058 (75)	2,066 (76)	0.80	1,544 (74)	763 (74)	781 (75)	0.37
Chronic kidney disease, n (%)	78 (1)	39 (1)	39 (1)	1.00	34 (2)	17 (2)	17 (2)	1.00
Atrial fibrillation, n (%)	86 (2)	43 (2)	43 (2)	1.00	62 (3)	31 (3)	31 (3)	1.00
Heart failure, n (%)	362 (7)	181 (7)	181 (7)	1.00	154 (7)	77 (7)	77 (7)	1.00
Initial Laboratory data								
Fasting blood glucose (mg/dl)	120 ± 45	119 ± 43	120 ± 46	0.43	108 ± 31	108 ± 31	108 ± 31	0.70
Total cholesterol (mg/dl)	229 ± 45	229 ± 47	229 ± 43	0.92	196 ± 44	196 ± 45	197 ± 43	0.51
Triglycerides (mg/dl)	287 ± 158	290 ± 162	285 ± 154	0.17	103 ± 29	104 ± 29	103 ± 29	0.97
LDL cholesterol (Martin-Hopkins)	137 ± 43	136 ± 46	137 ± 40	0.23	120 ± 42	120 ± 43	121 ± 41	0.53
LDL cholesterol (Friedewald)	123 ± 53	122 ± 56	124 ± 49	0.09	120 ± 44	120 ± 45	121 ± 42	0.54
HDL cholesterol (mg/dl)	48 ± 20	49 ± 22	48 ± 20	0.38	56 ± 16	56 ± 19	56 ± 13	0.88
Non-HDL-C (mg/dl)	181 ± 22	180 ± 21	181 ± 22	0.09	140 ± 23	140 ± 22	141 ± 23	0.31
TRL cholesterol (mg/dl)	44 ± 20	44 ± 11	43 ± 19	0.05	20 ± 4	20 ± 4	20 ± 4	0.91
Follow-up duration (months)	88 ± 28	89 ± 31	87 ± 24	0.01	92 ± 31	95 ± 36	89 ± 26	0.001
Follow-up Laboratory data								
Fasting blood glucose (mg/dl)	119 ± 34	118 ± 33	119 ± 36	0.48	110 ± 27	111 ± 58	109 ± 27	0.23
Total cholesterol (mg/dl)	201 ± 38	202 ± 37	200 ± 39	0.06	187 ± 35	190 ± 34	185 ± 35	0.01
Triglycerides (mg/dl)	257 ± 126	269 ± 132	245 ± 118	<0.01	142 ± 66	154 ± 77	131 ± 51	<0.01
LDL cholesterol (Martin-Hopkins)	116 ± 32	117 ± 32	117 ± 33	0.97	110 ± 31	111 ± 30	108 ± 31	0.03
HDL cholesterol (mg/dl)	49 ± 11	50 ± 12	49 ± 11	0.35	55 ± 12	55 ± 12	56 ± 12	0.07
Non-HDL-C (mg/dl)	152 ± 24	152 ± 23	151 ± 24	0.12	132 ± 28	135 ± 27	129 ± 28	<0.01
TRL cholesterol (mg/dl)	35 ± 14	36 ± 15	34 ± 14	<0.01	22 ± 6	24 ± 7	21 ± 5	<0.01
Change in Laboratory data								
Fasting blood glucose (mg/dl)	−0.18 ± 40	0.28 ± 40	−0.65 ± 40	0.39	4 ± 32	4 ± 34	3 ± 30	0.43
Total cholesterol (mg/dl)	−39 ± 53	−40 ± 55	−39 ± 51	0.83	−15 ± 49	−13 ± 49	−17 ± 49	0.03
Triglycerides (mg/dl)	−87 ± 168	−87 ± 186	−87 ± 148	0.97	21 ± 64	29 ± 71	12 ± 55	<0.01
LDL cholesterol (Martin-Hopkins)	−29 ± 51	−29 ± 53	−29 ± 48	0.82	−17 ± 46	−15 ± 47	−19 ± 46	0.06
HDL cholesterol (mg/dl)	2 ± 23	1 ± 24	2 ± 22	0.24	−0.2 ± 16	−1 ± 19	0 ± 13	0.10
Non-HDL-C (mg/dl)	−41 ± 24	−41 ± 23	−41 ± 24	1.00	−15 ± 14	−12 ± 13	−17 ± 14	<0.01
TRL cholesterol (mg/dl)	−12 ± 21	−12 ± 24	−12 ± 19	0.86	2 ± 8	3 ± 9	1 ± 7	<0.01
Medication								
Fenofibrate duration (months)	15 ± 13	15 ± 13	0.0 ± 0.0	<0.001	17 ± 17	17 ± 17	0 (0.0)	<0.001
Statin				0.03				0.56
Low/moderate, n (%)	5,025 (92)	2,491 (91)	2,534 (93)		1,919 (92)	956 (92)	963 (93)	
High, n (%)	429 (8)	236 (9)	193 (7)		157 (8)	82 (8)	75 (7)	
Plus ezetimibe, n (%)	636 (12)	363 (13)	273 (10)	0.01	203 (10)	111 (11)	92 (9)	0.16

Continuous variables were summarized as means ± standard deviations.

Matched, propensity score matched; LDL, low-density lipoprotein; TRL, Triglyceride-rich lipoprotein; HDL, high-density lipoprotein; low/moderate, low or moderate intensity statins; high, high intensity statin.

### Follow-up lipid profiles after propensity score matching

Follow-up lipid levels and changes from baseline are summarized in Table 2. During follow-up, individuals in the TRL cholesterol ≥ median group showed larger overall lipid reductions than those in the <median group, with mean triglyceride changes of −87 ± 168 mg/dl versus +21 ± 64 mg/dl and TRL cholesterol changes of −12 ± 21 mg/dl versus +2 ± 8 mg/dl, respectively. However, within each stratum, follow-up lipid levels were broadly similar between fenofibrate users and non-users.

### Association between fenofibrate use and ASCVD

Table 3 summarizes the associations between fenofibrate use and clinical outcomes according to baseline TRL cholesterol strata. In models adjusted for TRL cholesterol, fenofibrate use was associated with a lower risk of ASCVD among statin-treated individuals with baseline TRL cholesterol ≥ median, both in the pre-matched cohort (hazard ratio [HR] 0.82, 95% confidence interval [CI] 0.68–0.98) and after propensity score matching (HR 0.76, 95% CI 0.59–0.99). In contrast, no statistically significant association was observed in individuals with TRL cholesterol < median in either the pre-matched (HR 0.94, 95% CI 0.73–1.22) or post-matched analyses (HR 0.75, 95% CI 0.50–1.13).

**Table 3:** Risk of ASCVD and all-cause mortality in statin treated individuals with versus without fenofibrate use

Characteristics	TRL Cholesterol ≥ median			TRL Cholesterol < median		
	With	Without	HR (95% CI)	With	Without	HR (95% CI)
	Fenofibrate	Fenofibrate		Fenofibrate	Fenofibrate	
	no. of individuals with events/total no. (%)			no. of individuals with events/total no. (%)		
Adjusted by TRL cholesterol^a^						
ASCVD						
Pre-matched	151/3,228 (5%)	1,568/30,603 (5%)	0.82 (0.68–0.98)	72/1,271 (6%)	1,588/32,560 (5%)	0.94 (0.73–1.22)
Post-matched	113/2,727 (4%)	127/2,727 (5%)	0.76 (0.59–0.99)	48/1,038 (5%)	53/1,038 (5%)	0.75 (0.50–1.13)
All-cause mortality						
Pre-matched	162/3,228 (5%)	2,245/30,603 (7%)	0.37 (0.30–0.44)	94/1,271 (7%)	2,572/32,560 (8%)	0.28 (0.22–0.35)
Post-matched	115/2,727 (4%)	165/2,727 (6%)	0.51 (0.40–0.66)	66/1,038 (6%)	74/1,038 (7%)	0.57 (0.39–0.82)
Adjusted by triglyceride^b^						
ASCVD						
Pre-matched	151/3,228 (5%)	1,568/30,603 (5%)	0.82 (0.68–0.98)	72/1,271 (6%)	1,588/32,560 (5%)	0.94 (0.73–1.22)
Post-matched	113/2,727 (4%)	127/2,727 (5%)	0.77 (0.59–0.99)	48/1,038 (5%)	53/1,038 (5%)	0.75 (0.50–1.13)
All-cause mortality						
Pre-matched	162/3,228 (5%)	2,245/30,603 (7%)	0.37 (0.31–0.45)	94/1,271 (7%)	2,572/32,560 (8%)	0.28 (0.22–0.35)
Post-matched	115/2,727 (4%)	165/2,727 (6%)	0.51 (0.40–0.66)	66/1,038 (6%)	74/1,038 (7%)	0.57 (0.39–0.82)

Matched, propensity score matched; HR, hazard ratio; ASCVD, atherosclerotic cardiovascular disease.

a Adjusted for age, sex, body mass index, hypertension, diabetes, income tertile, cumulative low-density lipoprotein cholesterol, and cumulative TRL cholesterol.

b Adjusted for age, sex, body mass index, hypertension, diabetes, income tertile, cumulative low-density lipoprotein cholesterol, and cumulative triglyceride.

Similarly, in TRL cholesterol–adjusted analyses, fenofibrate use was associated with lower all-cause mortality. In individuals with TRL cholesterol ≥ median, HRs were 0.37 (95% CI 0.30–0.44) before matching and 0.51 (95% CI 0.40–0.66) after matching. Corresponding estimates in individuals with TRL cholesterol < median were 0.28 (95% CI 0.22–0.35) and 0.57 (95% CI 0.39–0.82), respectively.

When triglycerides were used instead of TRL cholesterol for covariate adjustment, results were materially unchanged. Triglyceride-adjusted models demonstrated a lower risk of ASCVD associated with fenofibrate use in individuals with baseline TRL cholesterol ≥ median (pre-matched HR 0.82, 95% CI 0.68–0.98; post-matched HR 0.77, 95% CI 0.59–0.99), but not in those with TRL cholesterol < median. Associations between fenofibrate use and lower all-cause mortality remained consistent in triglyceride-adjusted analyses across both TRL cholesterol strata (Table 3).

**Fig. 2:**
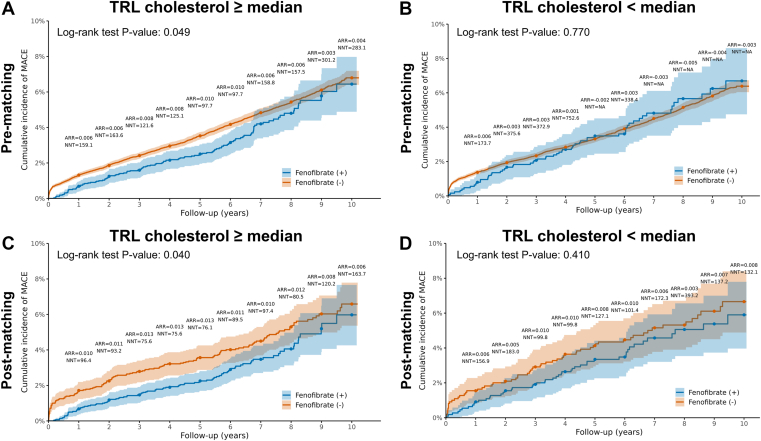
Kaplan-Meier survival plots of event free ASCVD survival for pre- and post-matched analyses in individuals with TRL cholesterol levels at or above versus below the median. Pre-matched analysis of individuals with TRL cholesterol ≥ median (A) and TRL cholesterol < median (B). Post-matched analysis of individuals with TRL cholesterol ≥ median (C) and TRL cholesterol < median (D). Matched, propensity score matched.

Kaplan–Meier analyses of the pre- (Fig. 2A, B) and post-matched cohorts (Fig. 2C, D) were consistent with these findings from the multivariable-adjusted models. In individuals with TRL cholesterol ≥ median, fenofibrate users had higher ASCVD event-free survival than non-users in the pre-matched cohort (Fig. 2A; log-rank *P* = 0.049) and in the post-matched cohort (Fig. 2C; log-rank *P* = 0.040). In individuals with TRL cholesterol < median, no significant separation of the Kaplan–Meier curves was observed either in the pre-matched cohort (Fig. 2B; log-rank *P* = 0.770) or in the post-matched cohort (Fig. 2D; log-rank *P* = 0.410).

ARR and NNT were estimated at follow-up time points based on cumulative incidence curves in pre- (Fig. 2A, B) and post-matched cohorts (Fig. 2C, D). After matching in the TRL cholesterol ≥ median group, ARR was greatest during the early follow-up period, with larger ARR and lower NNT values observed within the first 4–5 years (ARR approximately 1.3%, corresponding to NNT values of ∼75–76), followed by a gradual attenuation over longer follow-up (Fig 2A, C). In contrast, among individuals with TRL cholesterol < median, ARR values were small and inconsistent over follow-up, resulting in large or non-estimable NNT values (Fig. 2B, D).

### Sensitivity analyses

**Fig. 3:**
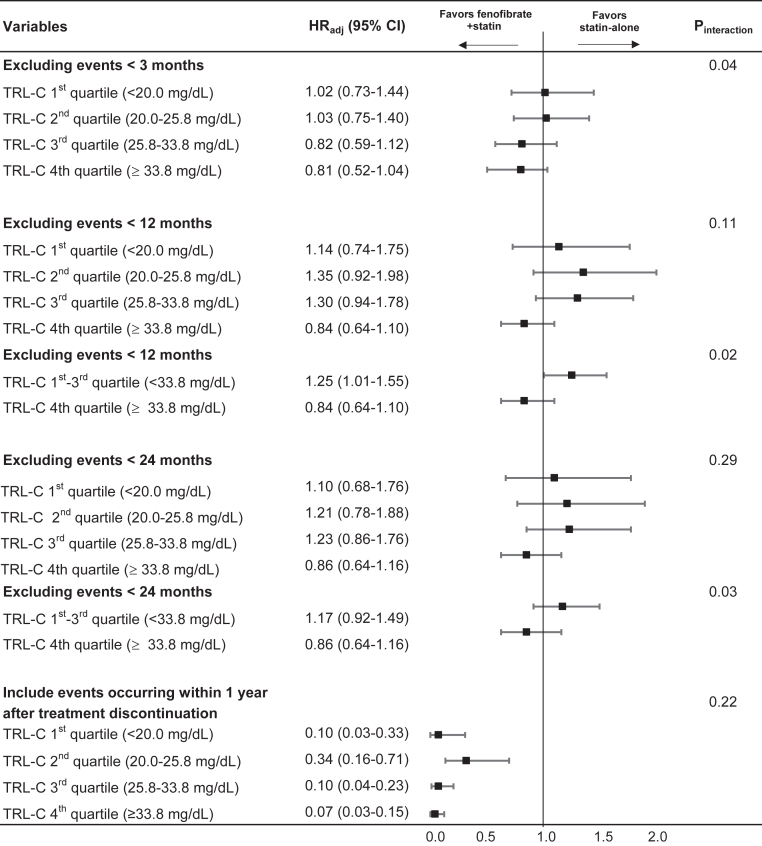
Sensitivity analyses of the association between fenofibrate use and ASCVD according to TRL cholesterol levels. HR_adj_; adjusted hazard ratio; RC, TRL cholesterol. Sensitivity analyses were performed using different analytic approaches, including exclusion of early events and alternative categorizations of TRL cholesterolHazard ratios were adjusted for age, sex, body mass index, hypertension, diabetes, income tertile, cumulative LDL cholesterol, and cumulative TRL cholesterol.

Sensitivity analyses examining the robustness of the association between fenofibrate use and ASCVD, including analyses designed to minimize potential reverse causality, are presented in Fig. 3. When ASCVD events occurring within the first 3 months after cohort entry were excluded, a graded association according to TRL cholesterol levels was observed, with lower hazard ratios in higher TRL cholesterol quartiles (P for interaction = 0.04). When events occurring within the first 12 months were excluded, the direction of association remained similar; however, the interaction across TRL cholesterol strata was attenuated and did not reach statistical significance (P for interaction = 0.11). When TRL cholesterol was dichotomized as the highest quartile versus lower quartiles, exclusion of early events within 12 months showed a significant interaction (P for interaction = 0.02). In analyses excluding events within the first 24 months, no statistically significant interaction according to TRL cholesterol strata was observed (P for interaction = 0.29). When TRL cholesterol was dichotomized as the highest quartile versus lower quartiles, exclusion within 24 months did show significant interaction (P for interaction = 0.03). In additional analyses allowing ASCVD events occurring within one year after fenofibrate discontinuation to be included, hazard ratios consistently favored fenofibrate use across all TRL cholesterol quartiles, with the strongest associations observed in the highest TRL cholesterol quartile (Fig. 3).

### Subgroup analyses by LDL and TRL cholesterol

**Fig. 4:**
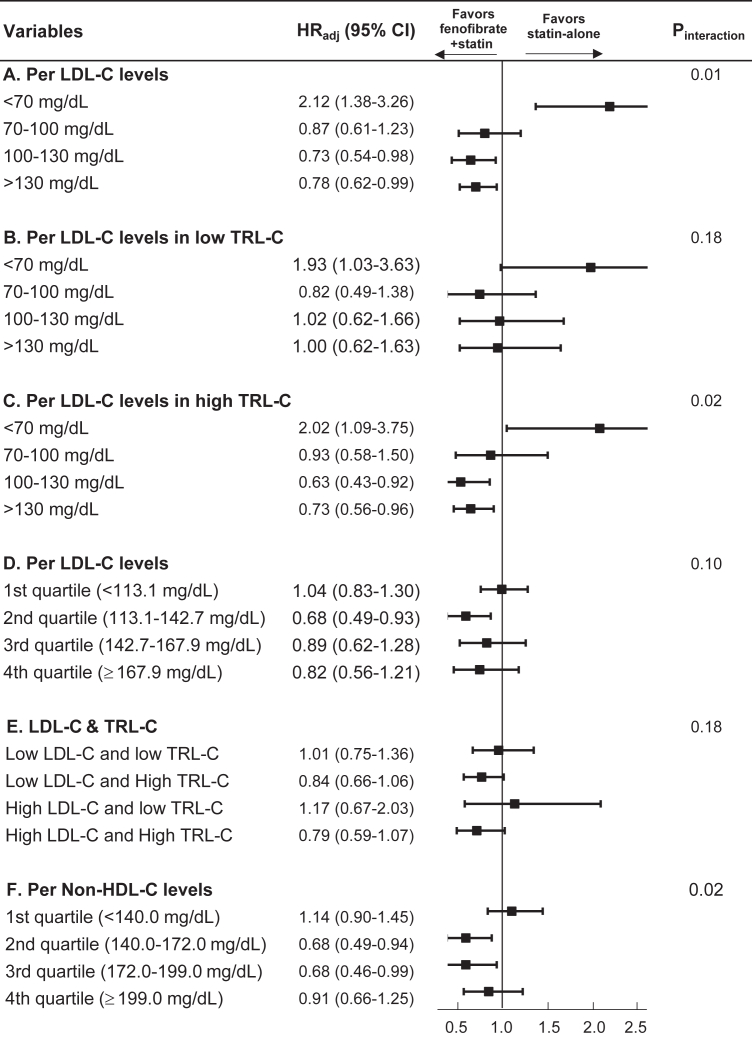
Subgroup analyses of ASCVD risk according to baseline LDL-C levels, TRL cholesterol levels, and non-HDL cholesterol. HR_adj_, adjusted hazard ratio; HDL, high-density lipoprotein.

Subgroup analyses according to baseline lipid parameters are shown in Figure 4. When stratified by baseline LDL cholesterol levels, a significant interaction was observed (P for interaction = 0.01), with fenofibrate use associated with lower ASCVD risk primarily among individuals with LDL cholesterol ≥100 mg/dl, whereas no evidence of benefit was observed in those with LDL cholesterol <70 mg/dl (Fig. 4A). When LDL cholesterol strata were further examined according to baseline TRL cholesterol levels, a significant interaction was observed in individuals with high TRL cholesterol (P for interaction = 0.02), but not in those with low TRL cholesterol (P for interaction = 0.18) (Fig. 4B, C). No significant interaction was observed when LDL cholesterol was analyzed by quartiles (P for interaction = 0.10; Fig. 4D) or when LDL cholesterol and TRL cholesterol were analyzed jointly (P for interaction = 0.18; Fig. 4E). However, across LDL cholesterol strata, lower hazard ratios associated with fenofibrate use were consistently observed in individuals with high TRL cholesterol, whereas no consistent benefit was seen in those with low TRL cholesterol.

### Subgroup analysis by non-HDL-C strata

When stratified by baseline non–HDL-C levels (Fig. 4F), the association between fenofibrate add-on therapy and lower ASCVD risk differed across quartiles (P for interaction = 0.02). A significant benefit was also observed in individuals with Non-HDL-C ≥140 mg/dl, with adjusted HR 0.68 (95% CI 0.49–0.94) for non–HDL-C 140–172 mg/dl and 0.68 (95% CI 0.46–0.99) for non–HDL-C 172–199 mg/dl n the highest quartile (≥199 mg/dl), although statistical significance was not reached (adjusted HR 0.91; 95% CI 0.66–1.25), a trend toward benefit was observed. In contrast, no benefit was observed in the lowest quartile (<140 mg/dl), with an adjusted HR of 1.14 (95% CI 0.90–1.45).

## Discussion

In 70,000 individuals from Korea without baseline ASCVD, fenofibrate added to statin compared to statin alone was associated with 24% lower risk of ASCVD in individuals with high TRL cholesterol. Importantly, this association remained directionally consistent across multiple sensitivity analyses and was most pronounced among individuals in the highest quartiles of baseline TRL cholesterol. A similar pattern was observed across non–HDL-C strata, with the benefit of fenofibrate being most evident among individuals with non–HDL-C levels ≥140 mg/dl, particularly in the third and fourth quartiles.

Previous major randomized controlled trials on fenofibrate have largely yielded modest or unsuccessful results. The FIELD trial recruited statin-naïve individuals with diabetes (N = 9,795), who had a total cholesterol to HDL cholesterol ratio ≥ 4.0 or triglyceride concentrations between 1.0 and 5.0 mmol/L (89 and 443 mg/dl), with 78% and 22% of individuals in the setting of primary and secondary prevention. (24) The primary endpoint was coronary events during 5 years. Although fenofibrate did not show a benefit for the primary endpoint overall, there were reductions in secondary endpoints like myocardial infarction, ASCVD, and coronary revascularization. The subsequent ACCORD study enrolled high-risk statin-treated individuals with diabetes (N = 5,518), with 64% and 36% of individuals in the setting of primary and secondary prevention. (26) That trial had no specific enrollment criteria for triglycerides, except an upper limit of <750 mg/dl. A key difference from the FIELD trial was that ACCORD included individuals already on a baseline statin therapy. From these two randomized controlled trials, we inferred that fenofibrate exhibited a positive signal for cardiovascular benefit in the FIELD trial, although this signal might have been diluted in the ACCORD study due to potent effects of statins on LDL cholesterol profiles. Importantly, in both trials there was a clear benefit of fenofibrate in individuals with high baseline TRL cholesterol (marked by high plasma triglycerides) but not in those with low levels, fully in accordance with the present observational finding including 70,000 statin-treated individuals in the primary prevention setting. Previous Korean real-world analyses have reported similar findings regarding fenofibrate use in statin-treated patients with atherogenic dyslipidemia. (38, 39, 40) Our results are broadly consistent with these studies. In our TRIUMPH cohort, the association appeared more evident among individuals with higher TRL cholesterol levels, although these observations should be interpreted cautiously.

Our data demonstrate that TRL cholesterol assessment is likely crucial for evaluating cardiovascular risk and predicting the effect of fenofibrate therapy in high-risk primary prevention settings treated with statins. One concern for the present data used is that LDL cholesterol is not routinely measured in Korea’s health check-up system (like in most other places in the world) unless triglycerides exceed 400 mg/dl. Accurate direct measurement of LDL cholesterol is important in these settings; however, when this is not feasible, using the Martin-Hopkins calculation (as done in the present study) is more accurate than the Friedewald equation. The Friedewald equation may overestimate TRL cholesterol or TRL cholesterol compared to using directly measured LDL cholesterol or the Martin-Hopkins equation, because Friedewald uses a fixed triglycerides/VLDL cholesterol ratio of 5. (32, 33) In contrast, the Martin-Hopkins calculation adjusts this ratio based on non-HDL cholesterol and triglycerides levels. This adjustable factor is approximately 5 when triglycerides are around 100 mg/dl, decreases to 3 with lower triglycerides, and increases up to 10 when triglycerides exceed 400 mg/dl. This background explains why, despite triglyceride levels being 90–110 mg/dl higher in individuals with high TRL cholesterol in the present TRIUMPH cohort compared to the previous FIELD and ACCORD trials, TRL cholesterol was only 6–9 mg/dl higher. If the Friedewald equation had been used for LDL cholesterol calculation in the present TRIUMPH cohort, the TRL cholesterol gap between the TRIUMPH cohort and the FIELD and ACCORD would have been 18–22 mg/dl. Therefore, when evaluating residual cardiovascular risk in statin-treated individuals in primary prevention settings, measuring LDL cholesterol as accurately as possible is essential. If direct measurement is not available, applying a more accurate LDL cholesterol calculation like the Martin-Hopkins method is recommended.

The lack of cardiovascular benefit in the Pemafibrate to Reduce Cardiovascular Outcomes by Reducing Triglycerides in Patients with Diabetes (PROMINENT) trial may reflect key mechanistic differences between pemafibrate and fenofibrate. (41) Pemafibrate is a highly potent and selective peroxisome proliferator-activated receptor-α (PPAR-α) agonist with approximately 2,500-fold greater activity than the active metabolite of fenofibrate (42), leading to stronger suppression of triglycerides and TRL cholesterol. However, this potency may have inadvertently induced modest increases in LDL cholesterol and apoB levels (41), likely due to enhanced lipolysis of VLDL particles combined with relatively inefficient clearance of the resulting LDL particles (43). In contrast, fenofibrate exerts more moderate PPAR-α activation, which appears to lower triglycerides without significantly raising LDL-C, particularly when used with statins. (44) Moreover, fenofibrate has been shown to reduce apoB-containing particle number, contributing to a more favorable atherogenic profile. (44) While both agents are generally compatible with statin therapy, fenofibrate’s more balanced lipid effects may help explain the discrepant cardiovascular outcomes observed between trials.

Fenofibrate’s lipid-modifying actions extend beyond simple triglyceride reduction and involve multiple pathways affecting TRL metabolism and HDL production. By activating PPAR-α, fenofibrate upregulates lipoprotein lipase and suppresses apolipoprotein C-III, which accelerates the catabolism of TRLs (e.g., VLDL and chylomicron remnants) into smaller cholesterol-rich remnant particles. (45, 46) This mechanism underlies the substantial reduction in plasma triglyceride levels (often ∼40% in hypertriglyceridemic patients) and the concomitant decrease in circulating remnant lipoproteins observed with fenofibrate therapy. Additionally, PPAR-α activation induces the synthesis of major HDL apolipoproteins (apolipoprotein A-I and apolipoprotein A-II), leading to a moderate increase in HDL cholesterol (approximately +10–15% in clinical studies). (45) The combined effect of lowering atherogenic particles, reducing triglyceride, and raising HDL is thought to contribute to fenofibrate’s cardiovascular benefit, which aligns with our findings in individuals with high TRL cholesterol despite statin therapy.

Interpretation of lipid changes over time requires particular caution. In the NHIS–NSC, follow-up lipid measurements are obtained during periodic national health examinations and are not synchronized with treatment initiation or discontinuation. As a result, these values often do not represent true on-treatment lipid levels, especially given the relatively short duration of fenofibrate use. Although we evaluated baseline, follow-up, and cumulative lipid parameters and incorporated them into multivariable models, the structural limitations of the dataset preclude definitive conclusions regarding treatment-mediated lipid changes. Accordingly, lipid trajectory analyses in this study should be considered descriptive rather than mechanistic.

Clinically, the present results are important. Because several fibrate trials overall have been negative (20, 24, 26, 41), particularly when given on a background of statin therapy (26, 41), many doctors are now less inclined to prescribe fibrate therapy on top of statins. Nevertheless, worldwide, it is still common to use fibrates in statin-treated individuals with residual elevated TRL cholesterol (marked by elevated triglycerides), which is particularly common in many parts of Asia, such as Korea, Japan, and India. (38, 39, 40, 47, 48) This strategy is supported by post-hoc analyses of many fibrate trials with and without statin therapy, showing that in individuals with elevated TRL cholesterol (marked by elevated triglycerides), fibrate therapy is indeed leads to reduced risk of ASCVD. (6, 25) The present findings likewise support this strategy, as using real-world data, we here show that fenofibrate added to statin therapy in individuals with high TRL cholesterol is associated with 24% lower risk of ASCVD in the primary prevention setting.

Non–HDL-C represents the total burden of atherogenic lipoproteins, including LDL, very-low-density lipoprotein, intermediate-density lipoprotein, and remnant lipoproteins. For this reason, several international guidelines recognize non–HDL-C as an important marker of residual atherogenic risk, particularly in individuals with hypertriglyceridemia. The 2019 ESC/EAS dyslipidemia guideline recommends specific non–HDL-C targets as secondary treatment goals (1), while the 2018 AHA/ACC cholesterol guideline highlights non–HDL-C as a useful marker of atherogenic lipoprotein burden beyond LDL-C. (49) In the present study, fenofibrate showed a consistent association with lower ASCVD risk among individuals with higher TRL cholesterol, with a pattern suggestive of a dose–response relationship across TRL-C quartiles (Fig. 3). In contrast, although a similar overall signal was observed across non–HDL-C strata, the dose–response relationship appeared less consistent, with attenuation of effect in the highest non–HDL-C quartile (Fig. 4F). However, our study was not specifically designed to directly compare the predictive performance of TRL cholesterol and non–HDL-C. Therefore, we cannot conclude that TRL cholesterol is superior to non–HDL-C in identifying individuals who benefit from fenofibrate therapy. Rather, the broadly concordant findings across both markers suggest that assessing the overall burden of atherogenic lipoproteins—whether estimated by TRL cholesterol or non–HDL-C—may help identify individuals who derive greater clinical benefit from fenofibrate add-on therapy.

### Limitations and strengths

The current study has some limitations. First, the Korean government does not provide direct LDL cholesterol measurement during national health checkups unless triglycerides exceed 400 mg/dl, thus necessitating estimated LDL cholesterol calculations. Also, the cohort size was limited as we used the NHIS-NSC cohort, which is “only” a 2% sample of the total Korean population. Third, the average duration of fenofibrate therapy was relatively short compared with the overall follow-up period, and adherence was inferred from prescription records rather than directly measured. Fourth, medication adherence was inferred from prescription records, assuming full coverage during follow-up, which may oversimplify actual refill behavior and drug-taking patterns and could have led to exposure misclassification. Fifth, on-treatment lipid levels could not be consistently assessed, as follow-up lipid measurements were obtained during periodic national health examinations rather than at predefined time points aligned with fenofibrate therapy. Consequently, many follow-up lipid values were collected after treatment discontinuation, limiting their ability to accurately reflect true on-treatment lipid responses. Sixth, although fenofibrate use was associated with lower all-cause mortality (38, 39, 40), this finding should be interpreted cautiously given the observational design and the potential for residual confounding, including healthy-user and treatment-selection effects. The magnitude of association exceeds that observed in randomized trials and therefore does not imply causality. Additionally, although our analyses focused on TRL cholesterol measured in fasting samples, it is possible that the observed association between fenofibrate therapy and ASCVD risk may partly reflect changes in remnant cholesterol carried within TRL particles. However, remnant cholesterol itself was not directly assessed in the present study. Finally, because the duration of fenofibrate therapy was substantially shorter than the overall follow-up period, post-discontinuation events were included in the primary analyses, which may have introduced survivor or exposure misclassification bias and warrant cautious interpretation of long-term associations.

It is a strength of our study that we obtained recruitment of participants unrelated to later use of statins or fenofibrate, or baseline TRL cholesterol levels. A further strength of the present study is that multiple sensitivity analyses were performed to minimize the potential impact of reverse causality, and across these analyses, a consistent direction of association between fenofibrate use and lower ASCVD risk was observed in individuals with higher TRL cholesterol.

## Conclusion

In this large, real-world cohort of statin-treated individuals without baseline ASCVD, fenofibrate use was associated with a modest reduction in ASCVD events among individuals with elevated baseline TRL cholesterol. These associations remained consistent across multiple sensitivity analyses, including adjustment for cumulative triglyceride levels and exclusion of early events. While causality cannot be inferred, the findings support a phenotype-guided approach to fibrate therapy, particularly in individuals with persistent TRL burden despite statin use.

## Data availability

The data that support the findings of this study are available from the Korean NHIS, but restrictions apply to the availability of these data, which were used under license for the current study, and so are not publicly available. Data may, however, be available upon reasonable request to the Korean NHIS (https://nhiss.nhis.or.kr/en/z/a/001/lpza001m01en.do).

## Conflict of interest

The authors declare the following financial interests/personal relationships which may be considered as potential competing interests:

BGN reports consultancies/talks for AstraZeneca, Sanofi, Regeneron, Ionis, Amgen, Kowa, Denka, Amarin, Novartis, Novo Nordisk, Esperion, Abbott, Silence Therapeutics, Ultragenyx, USV, Mankind, Lilly, Arrowhead, and Marea. Other authors have nothing to declare.
